# Micronutrient Supplementation for Pregnant and Lactating Women to Improve Maternal and Infant Nutritional Status in Low- and Middle-Income Countries: Protocol for a Systematic Review and Meta-analysis

**DOI:** 10.2196/40134

**Published:** 2022-08-30

**Authors:** Sachin Shinde, Dongqing Wang, Mashavu H Yussuf, Mary Mwanyika-Sando, Said Aboud, Wafaie W Fawzi

**Affiliations:** 1 T H Chan School of Public Health Harvard University Boston, MA United States; 2 Africa Academy for Public Health Dar es Salaam United Republic of Tanzania; 3 Department of Microbiology and Immunology Muhimbili University of Health and Allied Sciences Dar es Salaam United Republic of Tanzania

**Keywords:** antenatal care, multiple micronutrient supplementation, pregnant and lactating women, systematic review and meta-analysis, review, meta-analysis, meta-analyses, low- and middle-income countries, LMIC, low income, middle income, women's health, pregnant, pregnancy, natal, maternal, maternity, infant, baby, babies, lactation, lactating, breastfeed, nutrition, vitamin, nutrient, supplement

## Abstract

**Background:**

Two billion people in low- and middle-income countries (LMICs) are deficient in key nutrients. Nutritional deficiencies worsen during pregnancy, causing adverse outcomes for the mother and the fetus, with consequences after pregnancy. These effects may be mitigated by providing micronutrient supplementation to women during pregnancy and lactation. However, the effects of micronutrient supplementation on the nutritional status of pregnant and lactating women and that of their infants remain largely unclear in LMICs.

**Objective:**

The purpose of this systematic review and meta-analysis is to determine the effects of single, double, or multiple micronutrient supplements during pregnancy or lactation on maternal and infant nutritional status in LMICs.

**Methods:**

Randomized controlled trials of single, double, or combinations of micronutrients assessing effects on the maternal (serum, plasma, and breastmilk) and infant (serum and plasma) nutritional status will be included. MEDLINE (through PubMed), EMBASE, CENTRAL (through Cochrane Library), and the World Health Organization (WHO) library database will be used to identify relevant published studies, starting from the inception of each database until February 28, 2022. The Cochrane Risk of Bias Tool will be used to assess the risk of bias in the included studies. The selection of studies, data extraction, and risk of bias assessment will be carried out independently by 2 reviewers. A narrative summary will be provided of all the included studies. Meta-analyses will be performed whenever possible, and the heterogeneity of effects will be evaluated using I^2^, subgroup analyses, and metaregression. The certainty of the evidence for each outcome will be assessed using the GRADE (Grading of Recommendation, Assessment, Development, and Evaluation) approach.

**Results:**

We will conduct meta-analyses using Stata software (version 16, StataCorp) and present both a narrative and systematic summary of all studies included in this review in text and table form. For continuous outcomes, effect estimates will be expressed as mean differences and standardized mean differences, while for binary outcomes, they will be expressed as risk ratios, rate ratios, hazards ratios, or odds ratios, all with 95% CIs and comparing the intervention group with the control group. When studies for an outcome are adequately consistent with respect to intervention, comparator, and definition of the outcome, a random-effects, inverse variance-weighted meta-analysis will be conducted. We will provide a narrative synthesis for outcomes with insufficient data or extreme heterogeneity.

**Conclusions:**

This review will provide evidence upon which to base policy and programming for women in LMICs to supplement micronutrients in pregnancy and lactation. Detailed results disaggregated by variables such as maternal age, sex of infant, duration, and dose of intervention may also help policy makers, researchers, practitioners, and government agencies to adopt more effective maternal and child health policies and programs in LMICs. The review will also identify any gaps in the existing evidence.

**Trial Registration:**

PROSPERO CRD42022308715; https://tinyurl.com/y33cxekr.

**International Registered Report Identifier (IRRID):**

PRR1-10.2196/40134

## Introduction

### Background

Micronutrient deficiency is defined as an insufficient intake of vitamins and minerals required in small amounts by the body for good health, growth, and development [1]. Often referred to as hidden hunger, micronutrient deficiency, undernutrition, and overweight and obesity make up the triple burden of malnutrition. Worldwide, there are around 2 billion people who do not receive adequate micronutrients [2,3]. Women of reproductive age (15-49 years old) in low- and middle-income countries (LMICs) often have concurrent deficiencies of multiple micronutrients due to inadequate dietary intake and limited choices in fruits, vegetables, animal proteins, and fortified foods [1]. Micronutrient deficiencies can also be caused by infections and chronic diseases that directly interfere with nutrient absorption [4]. The World Health Organization (WHO) contends that micronutrients are important for maternal and child health because they help the body produce hormones, enzymes, and other substances essential for normal growth, development, and functionality [1]. In pregnancy and lactation, however, the burden and severity of micronutrient deficiencies are worsened by the increased demand, leading to potentially adverse effects on both the mother and her newborn [5]. Moreover, repeated pregnancies and short interpregnancy intervals may also affect maternal micronutrient status [6]. As a result, multiple micronutrient deficiencies are common among pregnant women, particularly in LMICs [7].

Anemia affects half a billion women worldwide or about 29% of nonpregnant women and 38% of pregnant women, mostly in South Asia and Central and West Africa, while maternal vitamin A deficiency affects approximately 15% of pregnant women [8]. It is well-established that iron-deficiency anemia in the reproductive age, especially during pregnancy, can lead to adverse maternal and newborn outcomes, including an increased risk of maternal mortality, perinatal mortality, and low birth weight [9]. Deficiencies in other micronutrients, such as vitamins A, B-complex, C, D, and E, are also common in LMICs, resulting in poor pregnancy outcomes, fetal growth retardation, and maternal and child health problems [9-13]. For example, folate deficiency is undoubtedly associated with neural tube defects [14], and low vitamin D levels during pregnancy may contribute to preeclampsia, small-for-gestational age, and perinatal mortality [15]. Malnutrition of the mother can also impact her offspring's long-term outcomes, such as growth, neurodevelopment, cognition, and cardiovascular, pulmonary, and immune function [7]. Therefore, unaddressed micronutrient deficiencies may threaten the survival and well-being of women and their newborns and put subsequent generations at risk of malnutrition due to intergenerational transfer [4].

Micronutrient malnutrition among women can be reduced by diet diversification, large-scale and targeted fortification, biofortification of staple crops, and micronutrient supplements [16]. Multivitamins and mineral supplements are commonly consumed during pregnancy in high-income countries, but this practice is less common in LMICs. Currently, there is a move toward multiple micronutrient supplementation in pregnant women to reduce adverse pregnancy outcomes in these parts of the world. To address the issue of multiple and concurrent micronutrient deficiencies, the United Nations Children’s Fund, United Nations University, and the WHO developed a multiple micronutrient (MMN) tablet called UNIMMAP (United Nations International Multiple Micronutrient Antenatal Preparation). The MMN tablet provides the daily recommended intakes of vitamins A (800 µg), B1 (1.4 mg), B2 (1.4 mg), B6 (1.9 mg), B12 (2.6 µg), C (70 mg), D (200 IU), E (10 mg), niacin (18 mg), folic acid (400 µg), copper (2 mg), selenium (65 µg), and iodine (150 µg) with 30 mg of iron and 15 mg of zinc for pregnant women. [17] However, the WHO does not yet universally recommend multiple micronutrient supplements for pregnant women over the current practice of prenatal supplementation with iron and folate, except in the context of rigorous research [18,19].

Most of the recent studies and reviews on maternal and infant nutrition and pregnancy outcomes have approached the issue by investigating either a single micronutrient or multiple micronutrients [9,10,14-16,20,21]. In many of these reviews, data from studies conducted in LMICs are included. However, significant heterogeneity in results has been reported, and it is unclear whether micronutrient supplementation has comparatively greater benefits in any particular settings or subgroups. Furthermore, while there is ample evidence of the benefits of maternal micronutrient supplementation on pregnancy outcomes [22], few studies have examined the effect of these supplements on micronutrient status among women and children. Many of the systematic reviews are several years old [16,20,21] and thus highlight the need for an updated synthesis of evidence based on more recently completed trials. Therefore, a synthesis of evidence on micronutrient supplementation during pregnancy and lactation, which focuses on vitamins and their effect on maternal and infant nutrition status in LMICs, will provide the basis for future research and discussions of policy implications.

**Specific Aims**

The review will summarize the available evidence on single, double, or multiple micronutrient supplements in LMICs. We aim to achieve the following objectives through this review: (1) to assess the effects of single, double, and multiple micronutrient supplements among pregnant or lactating women on maternal (plasma, serum, and breast milk) and infant nutritional status (plasma, and serum) in LMICs; (2) to understand the effect modifiers (such as micronutrient supplement dose and duration) that might alter the impact of micronutrient supplements on maternal and infant status; and (3) to identify subgroups of women and infants who might experience greater effects from micronutrient supplements and identify the sources of heterogeneity across studies.

## Methods

### Data Sources, Search Terms, and Search Strategy

We will search MEDLINE (through PubMed), EMBASE, CENTRAL (through Cochrane Library), and the WHO library database and conduct a manual search of bibliographies to identify potentially relevant published studies using a combination of medical subject headings (MeSH) and text words denoting micronutrient supplements and maternal and infant micronutrient status. We will search all databases for eligible studies starting with the launch of each database until February 28, 2022. We will also examine cross-references and bibliographies of the included studies to identify additional sources of information. This search will be supplemented by reviewing ClinicalTrials.gov and organizational websites such as the International Initiative for Impact Evaluations, WHO, World Bank, United Nations Children’s Fund, and the United Nations Population Fund. When possible, reports written in languages other than English will be translated by colleagues who are native speakers of those languages. Studies that cannot be adequately translated will not be considered.

We will use the PICO (participant, intervention, control, and outcomes) model (Table 1) to guide our search strategy, but we will not be restricted by the outcome to maintain a broad search. The search will use indexing terms, including MeSH terms, keywords, and free text words. First, a broad search strategy will be performed in PubMed without time restrictions; for example, type of study (randomized controlled trial) AND intervention (single, double, or multiple micronutrient supplements) AND population (pregnant and lactating women) AND setting (low- and middle-income countries). We will confirm the sensitivity of the search strategy by identifying several sentinel articles. The PubMed strategy provided in Multimedia Appendix 1 will be adapted to suit other databases. We will document the following details for each search: databases searched, date of search, search strategy (ie, subject headings and keywords, including whether terms are expanded or truncated and how they are combined), filters used, and the number of records retrieved. Additionally, a source will be provided for each publication identified through manual search (ie, name of the journal, website, conference proceedings, etc).

**Table 1:** Eligibility criteria for the systematic review and meta-analysis in PICO (participant, intervention, control, and outcomes) format.

Population			Pregnant or lactating women of any age and parity, living in a low or middle‐income country
Intervention			Single, double, and multiple vitamin supplementation (including micronutrient powders, tablets, syrups, and lipid-based micronutrient supplements)
Control			Author-defined (placebo, only iron supplement, only iron and folic acid supplement, or no care)
**Outcomes**			Micronutrient deficiencies
	Maternal outcomes	Vitamin A serum/plasma/breastmilk retinol Vitamin B1 serum/plasma thiamine Vitamin B2 serum/plasma riboflavin (flavoenzymes flavin mononucleotide and flavin adenine dinucleotide) Vitamin B3 serum/plasma (niacin and metabolite) Vitamin B5 serum/plasma pantothenic acid Vitamin B6 serum/plasma/breastmilk pyridoxal phosphate Vitamin B7 serum/plasma biotin Vitamin B9 folic acid or folate serum/plasma/breastmilk Vitamin B12 serum/plasma cobalamin Vitamin C plasma/serum/breastmilk Vitamin D serum/plasma (25-hydroxyvitamin D) Vitamin D breast milk (D3) Vitamin E serum/plasma/breastmilk tocopherol Vitamin K serum/plasma/breastmilk	
	Newborn outcomes	Vitamin A serum/plasma retinol Vitamin B1 serum/plasma thiamine Vitamin B2 serum/plasma riboflavin (flavoenzymes, flavin mononucleotide, and flavin adenine dinucleotide) Vitamin B3 serum/plasma (niacin and metabolite) Vitamin B5 serum/plasma pantothenic acid Vitamin B6 serum/plasma pyridoxal phosphate Vitamin B7 serum/plasma biotin Vitamin B9 folic acid or folate serum/plasma Vitamin B12 serum/plasma cobalamin Vitamin C plasma/serum/breastmilk Vitamin D serum/plasma (25-hydroxyvitamin D) Vitamin E serum/plasma tocopherol Vitamin K serum/plasma	

#### Eligibility

The inclusion and exclusion criteria for this study are listed in Textbox 1.

**Inclusion and exclusion criteria.**

**Inclusion criteria**

- Only randomized controlled trials (RCTs) will be included. Participants may be randomly assigned, individually or in clusters, to intervention and comparison groups. Crossover designs will be eligible for inclusion.
- Studies involving healthy pregnant or lactating women of any age and parity.
- Studies conducted in low- and middle-income countries, as defined by the World Bank in the 2021 [23].
- Studies of single (vitamins A, B-complex, C, D, E, or K), double, or multiple vitamin supplementation (containing at least three micronutrients) in the form of tablets, drops, syrup, or powder for pregnant or lactating women. We will also include trials of multiple micronutrient supplements that contain folate (vitamin B) with iron supplements (mineral). There will be no restrictions regarding the duration of exposure to the intervention, the provider of the intervention, and the frequency of the intervention (eg, daily or intermittent supplementation).
- Studies that examined the impact of lipid-based micronutrient supplements. We will, however, analyze the studies focusing on lipid-based micronutrient supplements separately, as these provide additional calories and nutrients that might have independent effects on outcomes of interest.
- Studies including a control group that received only iron, only iron and folic acid supplement, placebo, or no care
- Studies assessing the maternal and infant micronutrient status as the outcomes using biochemical tests.Table 1 provides the details on maternal and infant outcome definitions. International units will be used for all maternal and infant outcomes.
- Published articles as well as ongoing studies that have preliminary findings available.  
- We will not place any restrictions on the study year, language, sample size, or duration of the intervention. 

**Exclusion criteria**

- Quasi-experiment trials and nonrandomized controlled studies.
- Studies without a proper comparator intervention arm (eg, uncontrolled before-after studies).
- Observational studies such as cohort, case-control, and cross-sectional designs.
- Editorials, commentaries, opinions, and review articles. However, we will use review articles to identify additional original articles.
- Studies focused on populations with specific conditions (eg, populations with chronic or genetic diseases such as HIV, tuberculosis, or metabolic disorders).
- Studies that examined the impact of fortified food supplements.
- Studies focusing only on pregnancy outcomes such as low birth weight, preterm birth, small for gestational age birth, perinatal death, stillbirth, and neonatal death, or maternal and infant status of minerals, and those that do not report on nutritional status.

### Data Management

Records retrieved from electronic databases will be stored in EndNote X9 (Clarivate Analytics). Additionally, the records will be imported into Covidence (Veritas Health Innovation), an internet-based program that facilitates the streamlined management of systematic reviews. Duplicates will be detected and removed first by EndNote and then by Covidence.

### Study Selection

Studies for the title and abstract screening and full-text screening will be managed using Covidence. First, 2 reviewers (authors SS and MHY) will independently assess all the search results (ie, titles and abstracts) based on the inclusion and exclusion criteria to eliminate irrelevant studies. Then, the full-text screening will be conducted in duplicate according to the same inclusion/exclusion criteria. If there is a difference of opinion between the 2 reviewers, it will be resolved in discussion or by a third reviewer (author DW), if necessary. The inter-rater agreement will be measured by computing the raw percentage of agreement and Cohen κ coefficient. Following the PRISMA (Preferred Reporting Items for Systematic Reviews and Meta-Analyses) statement [24], a study flow diagram will be maintained ​​stating the specific reasons for exclusion. Journal titles and authors' names will not be concealed from reviewers.

### Data Extraction

The data of studies included in the review will be independently extracted and entered by the 2 reviewers. A data extraction form will be developed and then pilot-tested on 5 randomly selected studies. We will extract the following information: title, authors (first author and corresponding author), contact information of the corresponding author, journal (or source for unpublished reports), calendar year of publication, calendar year of intervention, country, source of funding, study design, sample size (for cluster RCTs, number of clusters for each group, and number of participants in each group), sample characteristics (eg, age, sex, socioeconomic status), intervention (including timing, duration, dosage, nutritional content, and cointerventions), measure of adherence, comparator/control, outcomes assessed, and main findings with point estimates and measures of variance (standard errors, 95% CIs, or *P* values). We will compile multiple reports from a single study, as there may be additional results in several reports. For missing information or inconsistent results across reports of a single study, we will contact the corresponding author via email for more accurate results or additional information. We will attempt to contact the author a maximum of 2 times. If we cannot resolve the data issue after this, we will analyze the available data and discuss the possible impact of the missing data.

### Quality Assessment

Two independent reviewers will conduct the risk of bias assessment. We will resolve any uncertainties or disagreements by discussion or by bringing in a third reviewer if necessary. In each included RCT, we will assess the risk of bias for each outcome reported instead of performing an analysis of the whole study. We will use the second version of the Cochrane Risk of Bias tool, known as RoB II, for RCTs [25]. This tool considers the following 5 domains: bias arising from the randomization process, bias due to deviations from intended interventions, bias due to missing outcome data, bias in the measurement of the outcome, and bias in the selection of the reported results. Each domain will be judged as having a “low risk of bias,” “high risk of bias,” or “some concerns.” An RCT will be considered as having a low risk of bias if its risk is low across the domains; high risk of bias if its risk is high in at least 1 of its domains, or some concerns for 3 or more domains that lower confidence in the results. We will consider an RCT to have some concerns if it raises some concerns in at least 1 domain but is does not have a high risk of bias for any domain. If necessary, we will contact the authors of the reports to obtain more information. A summary of our assessment of bias risk will be compiled into a table in which each judgment, along with the justification, will be outlined. We will also analyze the overall strength of the evidence for each outcome using the GRADE (Grading of Recommendations Assessment, Development, and Evaluation) tool [26].

### Data Synthesis

A narrative and systematic summary of all the studies included in this review will be presented in the text as well as a table. We will treat micronutrient supplementation as a dichotomous exposure (intervention versus control). In the case of an intervention study with more than 2 arms, each arm will be treated separately. Effect estimates for continuous outcomes will be expressed as mean differences (MDs) and standardized mean differences (SMDs) with 95% CIs comparing the intervention group with the control group. Using the same format for each outcome (eg, means and standard deviations for continuous data), we will convert scales so that an increase/decrease always indicates improvements or deteriorations of an indicator. In cases where the data reported by included studies are not usable (ie, cannot be pooled with other data), we will contact the corresponding author for access to data or revised statistics. If we are unable to contact the corresponding author, or the data is unavailable, we will retain the study as eligible but restrict further analysis. Data on continuous outcomes will be presented as either an MD if outcomes have been measured on the same scale or an SMD if outcomes have been measured on different scales with 95% CIs. Changes in baseline scores and final measurements will be eligible for pooling if the scales and measurements are similar. Due to the differences in measurement reliability, we will not combine final values and change scores as SMDs. The standard deviation of the change will also be reported when combining measures of treatment effect with SMDs.

Effect estimates for binary outcomes will be expressed as risk ratios, rate ratios, hazard ratios, or odds ratios, all with 95% CIs and comparing the intervention group with the control group. Different studies may define deficiency of the same vitamin using different cutoffs. In the primary analyses, we will follow the cutoffs chosen by the authors to define the deficiency, while in the sensitivity analyses, we will restrict to only those studies that used well-established cutoffs. Our data extraction for RCTs will be based on intent-to-treat analyses. 

We will conduct a random-effects, inverse variance-weighted meta-analysis for an outcome if studies for that outcome are sufficiently consistent in terms of intervention, comparator, and outcome definition. Since the effect of micronutrient supplementation is expected to be heterogeneous across dose, duration, and populations, the random-effects method will be used. The generic inverse-variance approach will be used for both binary and continuous outcomes to adjust study weights according to the variance of the effect estimate. We will interpret overall effect estimates that have an associated *P* value less than 0.05 as statistically significant but will also comment on those effects where the upper and lower CIs have just crossed the line of no effect. The GRADE tool [26] will be used to assess the quality of the evidence for the outcomes for which the meta-analysis is to be conducted. Meta-analyses will be conducted only when there are data for a minimum 2 studies per the outcome of interest. Sensitivity analyses will be conducted to determine whether the removal of studies with a high risk of bias significantly influences findings.

We will assess effect heterogeneity by computing the I^2^ statistic, which represents the percentage of the total variation in the effect estimates that is due to true heterogeneity rather than chance. An I^2^ statistic over 50% will be considered substantial heterogeneity. We will assess the sources of heterogeneity by conducting subgroup analyses. Subgroup analyses for outcomes will be conducted when 2 or more studies are available per subgroup of interest. The following prespecified subgroups will be considered: pregnancy versus lactation status of women, maternal age, type/formulation of micronutrient supplement (single, double, or multiple micronutrients), duration of intervention, dosage of the micronutrient supplements, presence of cointerventions (by itself or combined with complementary interventions), baseline nutritional status in mothers, sex of infant, country or geographic region, and risk of bias (low, high, or some concerns). To further explain heterogeneity, we will perform a metaregression using the predictors mentioned above.

We will use contour-enhanced funnel plots to detect publication bias if there are 10 or more studies available for an outcome. Publication bias is unlikely if data forms a symmetric inverted funnel shape around the mean effect estimate. In addition, we will perform the Egger test to determine funnel plot asymmetry [27].

For outcomes with insufficient data or extreme heterogeneity that cannot be assessed in subgroup analyses or metaregression, we will provide a narrative synthesis without a meta-analysis. Statistical analyses will be conducted using Stata software (version 16, StataCorp).

### Registration and Reporting

This systematic review and meta-analysis protocol has been registered on the PROSPERO database (registration number CRD42022308715). In the event of protocol amendments, the date of each amendment will be accompanied by a description of each change and the rationale on PROSPERO. ​In preparing this protocol, we followed the PRISMA-P (Preferred Reporting Items for Systematic Reviews and Meta-analysis Protocols) checklist, [24], which is contained in Multimedia Appendix 2. ​Our systematic review will be reported following the Cochrane Handbook for Systematic Reviews of Interventions [28] as well as the PRISMA guidelines [24].

### Ethics Approval

Ethics approval was not required for this systematic review and meta-analysis because we are only examining secondary data already available in scientific databases.

## Results

As of June 30, 2022, we have run and imported the searches into all selected databases. In total, we have extracted and screened the 23,702 searches for titles and abstracts. We plan to complete the data extraction and analysis for selected studies and write the report by December 2022.

## Discussion

### Summary

In this planned review and meta-analysis, we will assess the evidence available on the impact of maternal multiple micronutrient supplements on infant and maternal nutrition. By synthesizing evidence regarding potential effect modifiers that could alter the effectiveness of micronutrient supplements on maternal and infant status, the findings from this study will fill important knowledge gaps and provide directions for future research and policies.

Appropriate care and adequate prenatal preparation and nutrition are important factors that affect the nutritional status and outcome of pregnancy for mothers, children, families, and society. Nutritional deficiency during pregnancy and lactation may have adverse effects on the mother, the child, and future generations. The prevalence of concurrent deficiencies of multiple micronutrients among pregnant women and young children is well documented, particularly in LMICs [5-8]. Micronutrient supplementation, either alone or in combination, has shown to be effective in improving maternal, birth, and child outcomes. For example, the 2019 Cochrane review of 17 trials found that multiple micronutrient supplementation with iron and folic acid after 20 weeks of pregnancy reduced preterm births and small-for-gestational-age births in underweight women, decreased small for-gestational-age births in normal-weight and normal-stature women, and reduced perinatal mortality when supplementation was initiated after 20 weeks of gestation [20]. However, there are still significant gaps in the evidence regarding the optimal dose of iron (30 versus 60 mg) and the timing and duration of multiple micronutrient supplements for maximum positive effects, as well as the extent and potential benefits of multiple micronutrients beyond anemia and pregnancy outcomes [29].

The WHO-recommended dose of iron ranges from 30 mg to 60 mg, although most prenatal programs have used a daily dose of 60 mg [18,19]. In contrast, the lower dose of 30 mg iron is included in the UNIMMAP preparation (together with 14 other micronutrients) [17], as the absorption of iron is expected to be enhanced due to vitamins C, A, and B2. There is growing scientific consensus that multiple micronutrient supplements containing iron and folic acid are superior to iron and folic acid supplementation alone. Recent data from an individual patient data meta-analysis of 17 RCTs including over 100,000 women living in LMICs found that multiple micronutrient supplementation in pregnancy reduced the risk of low birth weight, preterm birth, and being born small for gestational age [22,30]. Yet, a sensitivity analysis of 11 of these 17 trials showed that multiple micronutrient supplements containing low dose iron (≤30 mg) were associated with higher stillbirth and neonatal mortality than iron-folic acid alone with 60 mg iron [22,30]. Furthermore, in a meta-analysis of randomized trials of prenatal iron use, a dose-response analysis showed a linear decrease in maternal anemia with higher doses of iron, up to 66 mg per day. The meta-analysis also found an association between higher doses of iron with a linear increase in birth weight and a decrease in the risk of low birth weight [9].

Accordingly, WHO recommendations call for research on the use of multiple micronutrient supplementation over iron and folic acid supplementation alone for pregnant women in low-resource settings [18,19]. Moreover, the Micronutrient Forum recommends multimicronutrient supplementation as “context specific-research,” including the use of micronutrient supplements in the context of prenatal care services informed by implementation research as well as continuing clinical research as part of a global agenda to inform future WHO guidelines as they are revised and updated [31,32]. While current evidence from multiple micronutrients supplementation trials suggest the immediate benefit for pregnancy outcomes [16,18-20,22], future studies are warranted to examine the appropriate dose of multiple micronutrient supplementation and identify high-risk groups and regions where the effectiveness of prevention is likely to be the highest and thus may offer the greatest public health return on investment.

Multimicronutrient supplements can lower maternal morbidity and mortality by directly treating a pregnancy-related illness or by indirectly reducing complications during delivery when compared to iron and folic acid supplementation alone. However, the effectiveness of micronutrient supplementation programs has frequently been measured by the outcomes of pregnancy (eg, preterm birth, low birth weight, and perinatal mortality), and evidence regarding the effects of micronutrient supplementation on maternal and infant nutritional status is limited and inconsistent. In fact, in some cases, micronutrient deficiency was persistent even after multiple micronutrient supplementation. For instance, in a double-blind RCT in Nepal, zinc or zinc combined with other vitamins and minerals had no additional benefit, compared with iron plus folic acid, for improving iron status or anemia among pregnant women [33]. There have also been reports of persistent micronutrient deficiency in Bangladesh despite prenatal supplementation with multiple micronutrients promoting pregnancy-related benefits [34,35]. A clustered-randomized trial assessed the efficacy of a daily multiple micronutrient supplement with 15 nutrients, each provided approximately 1 recommended daily allowance (RDA). Compared with iron and folic acid supplement, vitamins B12, A, and D, and zinc status indicators were 3.7% to 13.7% higher, and ferritin, γ-tocopherol, and thyroglobulin indicators were 8.7% to 16.6% lower for the multiple micronutrient group compared with the iron and folic acid supplement group, with a 15% to 38% lower prevalence of deficiencies of vitamins B12, A, and D and zinc (*P*<.05 for all) [35]. Consequently, evaluating the effectiveness of multimicronutrient supplements and their dose in improving the nutritional status of both mothers and infants can help inform future formulations. 

Currently, there is only limited evidence available on the dosing of multiple micronutrient supplements. A double-blind, randomized controlled trial assessed the effects of prenatal multiple micronutrient supplementation with 1 or 2 RDA, compared with the conventional iron and folic acid supplementation, on birth weight and perinatal mortality in urban Guinea-Bissau. A dose-response effect was observed with a 53-gm increase in birth weight after administration of 1 RDA of the micronutrients and 95 gm after 2 RDA, and no differences were observed in perinatal mortality between the study groups [36]. In addition, there is mixed evidence on the effect of multimicronutrient supplements with varying dosages in HIV-infected pregnant women. For example, a 2 × 2 factorial design trial of vitamin A (30 mg β-carotene plus 5000 IU preformed vitamin A) and multivitamins (20 mg B1, 20 mg B2, 25 mg B6, 100 mg niacin, 50 μg B12, 500 mg C, 30 mg E, and 0.8 mg folic acid) in Tanzania showed that the provision of multivitamin supplements decreased the risk of fetal death, low birth weight, preterm birth, and small size for gestational age, whereas vitamin A supplementation did not [37]. There are studies in progress assessing the scaling up of multimicronutrient supplements, as the UNIMMAP formulation in Bangladesh, Madagascar, Burkina Faso, and Tanzania that might show substantial benefits in terms of mortality reduction and poor birth outcome, by shifting from iron folic acid supplementation to multimicronutrient supplement in prenatal care programs [29].

In addition, vitamin B12 deficiency has been linked to poor fetal growth and development and heightened chronic disease risks [7]. Among Indian women with an approximately 50% prevalence of B12 deficiency, prenatal 50 µg vitamin B12 supplements, nearly 20 times the dose in the UNIMMAP multiple micronutrient supplements, prevented a decline in vitamin B12 from early to late pregnancy [38]. In a blinded, placebo-controlled trial among Bangladeshi women randomized to receive 250 μg/day B12 (ie, 96-100-fold higher than RDA) or placebo throughout pregnancy and the 3-month postpartum period along with 60 mg iron and 400 μg folate, the maternal status of plasma vitamin B12 and colostrum and breast milk B12 was substantially improved by 250 μg/day supplements throughout pregnancy and lactation. In addition, infants born to supplemented mothers had improved vitamin B12 status (ie, higher plasma B12 and lower plasma homocysteine and methylmalonic acid concentrations) [39]. However, a randomized, double-blind, placebo-controlled trial assessing the effect of a multivitamin supplement on perinatal outcomes in Dar es Salaam, Tanzania, found that multivitamin supplements containing 50 µg of vitamin B12 produced a nonstatistically significant decline in the odds of having inadequate vitamin B12 in breast milk [40].

Consequently, there is a need to systematically synthesize the evidence to decide which micronutrients are of greatest concern in LMICs, the criteria for prophylactic single nutrient or multiple micronutrient supplementation, and the outcomes to be measured. We anticipate that the findings of this review will help advance the recommendations for scaling up multiple micronutrient supplementation during pregnancy [17,18,41]. Disaggregated results by other variables, such as maternal age, sex of infant, duration of intervention, and dose of intervention, may also aid policy makers, researchers, practitioners, and governmental and nongovernmental agencies in supporting better maternal and child health programs and policies in LMICs.

### Limitations

Our study has a couple of limitations. First, the data from this review will be gathered exclusively from published studies, excluding data from unpublished studies and or literature that has not been peer-reviewed (eg, reports). Although it has been suggested that researchers should aim to include unpublished studies in meta-analyses and systematic reviews, including these studies can in itself introduce bias [28]. It is likely that the unpublished studies that are located are not representative of all unpublished studies. In some cases, the identification of unpublished studies may be dependent on the willingness of investigators to provide data. This could depend again on the results of the study, with more favorable results being provided more readily [28]. Additionally, unpublished studies may have a lower methodological quality than published studies. Second, we will only collect data from RCTs, as we believe databases from LMICs contain enough randomized trials to answer the question of interest. Moreover, we will not include nonRCTs because their potential biases are likely to be greater when compared with RCTs. The nonrandomized studies of interventions also differ in their ability to estimate causal effects. 

## Appendix

Multimedia Appendix 1 PubMed search strategy.

Multimedia Appendix 2 PRISMA-*P* 2015 Checklist.

## Abbreviations

GRADE: Grading of Recommendation, Assessment, Development, and Evaluation
LMIC: low- and middle-income country
MD: mean difference
MeSH: medical subject heading
MMN: multiple micronutrient
PICO: participant, intervention, control, and outcomes
PRISMA: Preferred Reporting Items for Systematic Reviews and Meta-Analyses
PRISMA-P: Preferred Reporting Items for Systematic Reviews and Meta-analysis Protocols
RCT: randomized controlled trial
RDA: recommended daily allowance
SMD: standardized mean difference
UNIMMAP: United Nations International Multiple Micronutrient Antenatal Preparation
WHO: World Health Organization

## Data Availability

All data that will be generated and analyzed during this study will be included in the published article or its supplementary information files. We will submit our findings for peer-reviewed publication and present them at relevant conferences.
