# Supplementary material for: Micronutrient Supplementation for Pregnant and Lactating Women to Improve Maternal and Infant Nutritional Status in Low- and Middle-Income Countries: Protocol for a Systematic Review and Meta-analysis
Source: JMIR Res Protoc. 2022 Aug 30;11(8):e40134. doi: 10.2196/40134 (PMC9472043; doi:10.2196/40134)
Supplement: Multimedia Appendix 1 [file resprot_v11i8e40134_app1.docx]

**Multimedia Appendix 1: PubMed search strategy**

| **No.** | **Concept** | **Search terms** |
| --- | --- | --- |
| #1 | Trials | ‘Clinical Trials as Topic’[Mesh Terms] OR ‘Randomized Controlled Trial’[pt] OR Clinical Trial[pt] OR ‘Controlled Clinical Trial’[pt] OR ‘randomized controlled trials as topic’[MeSH terms] OR ‘controlled trial’ OR intervention OR ‘random allocation’[MeSH terms] OR random OR trial OR ‘Clinical Study’[pt] OR ‘Clinical Studies as Topic’[Mesh terms] OR ‘Therapeutic Uses’[Mesh Terms] OR ‘therapeutic use’[Subheading] |
| #2 | Intervention | ‘micronutrients’ [MeSH Terms] OR ‘micronutrients’ [All Fields] OR ‘micronutriments’ [All Fields] ‘micronutrient’ [All Fields] OR (‘dietary’ [All Fields] AND ‘supplements’ [All Fields]) OR ‘dietary supplements’ [All Fields] OR ‘supplement’ [All Fields] OR ‘supplements’ [All Fields] OR ‘supplemented’ [All Fields] OR ‘supplementing’ [All Fields] OR ‘supplement s’ [All Fields] OR ‘vitamins’ [MeSH Terms] OR ‘vitamins’ [All Fields] OR ‘vitamin’ [All Fields] OR ‘multi-vitamin’ [All Fields] OR ‘multivitamin’ [All Fields] OR ‘multivitamins’ [All Fields] OR ‘vitamin a’ [MeSH Terms] OR ‘vitamin a’ [All Fields] OR ‘retinol’ [All Fields] OR ‘retinols’ [All Fields] OR ‘vitamin b complex’ [MeSH Terms] OR ‘vitamin b complex’ [All Fields] OR ‘vitamin b’ [All Fields] OR ‘B-complex’ [All Fields] OR ‘thiamine’ [MeSH Terms] OR ‘thiamine’ [All Fields] OR ‘thiamin’ [All Fields] OR ‘thiamines’ [All Fields] OR (‘vitamin’ [All Fields] AND ‘b1’ [All Fields]) OR ‘vitamin b1’ [All Fields] OR ‘riboflavin’ [MeSH Terms] OR ‘riboflavin’ [All Fields] OR (‘vitamin’ [All Fields] AND ‘b2’ [All Fields]) OR ‘vitamin b2’ [All Fields] OR ‘niacinamide’ [MeSH Terms] OR ‘niacinamide’ [All Fields] OR ‘niacin’ [Mesh Terms] OR ‘niacin’ [All Fields] OR (‘vitamin’ [All Fields] AND ‘b3’ [All Fields]) OR ‘vitamin b3’ [All Fields] OR ‘pantothenic acid’ [MeSH Terms] OR (‘pantothenic’ [All Fields] AND ‘acid’ [All Fields]) OR ‘pantothenic acid’ [All Fields] OR (‘vitamin’ [All Fields] AND ‘b5’ [All Fields]) OR ‘vitamin b5’ [All Fields] OR ‘pyridoxine’ [MeSH Terms] OR ‘pyridoxine’ [All Fields] OR ‘pyridoxine’ [All Fields] OR ‘pyridoxines’ [All Fields] OR (‘vitamin’ [All Fields] AND ‘b6’ [All Fields]) OR ‘vitamin b6’ [All Fields] OR ‘biotin’ [MeSH Terms] OR ‘biotin’ [All Fields] OR (‘vitamin’ [All Fields] AND ‘b7’ [All Fields]) OR ‘vitamin b7’ [All Fields] OR ‘folic acid’ [MeSH Terms] OR (‘folic’ [All Fields] AND ‘acid’ [All Fields]) OR ‘folic acid’ [All Fields] OR ‘folate’ [All Fields] OR ‘folates’ [All Fields] OR (‘vitamin’ [All Fields] AND ‘b9’ [All Fields]) OR ‘vitamin b9’ [All Fields] OR ‘vitamin b 12’ [MeSH Terms] OR ‘vitamin b 12’ [All Fields] OR ‘cyanocobalamin’ [All Fields] OR ‘cyanocobalamine’ [All Fields] OR ‘ascorbic acid’ [MeSH Terms] OR (‘ascorbic’ [All Fields] AND ‘acid’ [All Fields]) OR ‘ascorbic acid’ [All Fields] OR ‘vitamin c’ [All Fields] OR ‘cholecalciferol’ [MeSH Terms] OR ‘cholecalciferol’ [All Fields] OR ‘cholecalciferols’ [All Fields] OR ‘vitamin d’ [MeSH Terms] OR ‘vitamin d’ [All Fields] OR ‘calcifediol’ [MeSH Terms] OR ‘calcifediol’ [All Fields] OR ‘25-hydroxyvitamin D’ [All Fields] OR ‘ 25 hydroxyvitamin d’ [All Fields] OR ‘ergocalciferols’ [MeSH Terms] or ‘ergocalciferols’ [All fields] OR ‘vitamin e’ [MeSH Terms] OR ‘vitamin e’ [All Fields] OR ‘vitamin k 1’ [MeSH Terms] OR ‘vitamin k 1’ [All Fields] OR ‘phytomenadione’ [All Fields] |
| #3 | Pregnant women and reproductive age women | (((‘gravidity’[MeSH Terms] OR ‘gravidity’[All Fields] OR ‘pregnant’ [All Fields] OR ‘pregnants’ [All Fields] OR ‘pregnant women’ [MeSH Terms] OR (‘pregnant’ [All Fields] AND ‘women’ [All Fields]) OR ‘pregnant women’ [All Fields]) AND ‘pregnancy’ [MeSH Terms]) OR ‘pregnancy’ [All Fields] OR ‘gestation’ [All Fields] OR ‘gestational’ [All Fields] OR (‘gestate’ [All Fields] OR ‘gestated’ [All Fields] OR ‘gestates’ [All Fields] OR ‘gestating’ [All Fields] OR ‘gestational’ [All Fields] OR ‘gestations’ [All Fields] OR ‘pregnancy’ [MeSH Terms] OR ‘pregnancy’ [All Fields] OR ‘gestation’ [All Fields]) OR ‘mothers’ [MeSH Terms] OR ‘mothers’ [All Fields] OR ‘mother’ [All Fields] OR ‘mothering’ [All Fields] OR ‘mothered’ [All Fields] OR ‘maternal’ [All Fields] OR ‘maternally’ [All Fields] OR ‘maternity’ [All Fields] OR ‘maternities’ [All Fields] OR ‘maternal age’ [MeSH Terms] OR (‘maternal’ [All Fields] AND ‘age’ [All Fields]) OR ‘maternal age’ [All Fields] OR ‘child bearers’ [All Fields] OR ‘childbearing’ [All Fields] OR ‘prenatal care’ [MeSH Terms] OR (‘prenatal’ [All Fields] AND ‘care’ [All Fields]) OR ‘prenatal care’ [All Fields] OR ‘perinatal care’ [MeSH Terms] OR (‘perinatal’ [All Fields] AND ‘care’ [All Fields]) OR ‘perinatal care’ [All Fields] OR ‘anti-natal’ [All Fields] OR ‘antenatal’ [All Fields] OR ‘pre-partum’ [All Fields] OR (‘pre’ [All Fields] AND ‘partum’ [All Fields]) OR ‘perinatology’ [MeSH Terms] OR ‘perinatology’ [All Fields] OR ‘peri-partum’ [All Fields] OR (‘peri’ [All Fields] AND ‘partum’ [All Fields]) OR ‘lactating’ [All Fields] OR ‘lactation’ [MeSH Terms] OR ‘lactation’ [All Fields] OR ‘breast feeding’ [MeSH Terms] OR (‘breast’ [All Fields] AND ‘feeding’ [All Fields]) OR ‘breast feeding’ [All Fields] OR ‘lactational’ [All Fields] OR ‘lactations’ [All Fields] OR ‘lactators’ [All Fields]) AND (‘women’ [All Fields] OR ‘women’ [MeSH Terms] OR ‘women’ [All Fields] OR ‘woman’ [All Fields] OR (‘reproductive’ [All Fields] AND ‘age’ [All Fields] AND ‘women’ [MeSH Terms] OR ‘women’ [All Fields] OR ‘woman’ [All Fields])) |
| #4 | Low- and middle-income countries | ‘Developing Countries’[MeSH Terms] OR ‘developing country’ OR ‘developing nation’ OR ‘less developed country’ OR ‘less developed nation’ OR ‘third world nation’ OR ‘third world country’ OR ‘under developed nation’ OR ‘underdeveloped nation’ OR ‘under developed country’ OR ‘underdeveloped country’ OR ‘middle income country’ OR ‘middle-income country’ OR ‘middle income nation’ OR ‘middle-income nation’ OR ‘low income country’ OR ‘low-income country’ OR ‘low income nation’ OR ‘low-income nation’ OR ‘poor country’ OR ‘poor nation’ OR LMIC OR lmics OR ‘Africa’[MeSH] OR ‘Asia’[MeSH] OR ‘South America’[MeSH Terms] OR ‘Latin America’[MeSH terms] OR ‘Central America’[MeSH Terms] OR Africa OR Asia OR ‘south America’ OR ‘Latin America’ OR ‘central America’ OR Afghanistan OR Albania OR Algeria OR Samoa OR Angola OR Armenia OR Azerbaijan OR Bangladesh OR Bengali OR Belarus OR Belize OR Benin OR Bhutan OR Bolivia OR Bosnia OR Herzegovina OR Botswana OR Brazil OR Bulgaria OR ‘Burkina Faso’ OR Burkinabe OR Burundi OR ‘Cabo Verd’ OR ‘Cape Verd’ OR Cambodia OR Cameroon OR ‘Central African’ OR Chad OR China OR Chinese OR Colombia OR Comoros OR Congo OR ‘Costa Rica’ OR ‘Cote d'Ivoire’ OR ‘Ivory Coast’ OR Cuba OR Cuban OR Djibouti OR Dominica OR Ecuador OR Egypt OR ‘El Salvador’ OR Eritrea OR Ethiopia OR Fiji OR Gabon OR Gambia OR Georgia OR Ghana OR Grenada OR Guatemala OR Guinea OR Guyana OR Haiti OR Honduras OR India OR Indian OR Indonesia OR Iran OR Iraq OR Jamaica OR Jordan OR Kazakh OR Kenya OR Kiribati OR ‘People's Republic of Korea’ OR ‘North Korea’ OR Kosovo OR Kosovar OR Kyrgyz OR Lao OR Laos OR Laotian OR Lebanon OR Lebanese OR Lesotho OR Liberia OR Libya OR Macedonia OR Madagascar OR Malawi OR Malaysia OR Maldives OR Mali OR ‘Marshall Island’ OR Mauritania OR Mexico[MeSH] OR Mexico OR Mexican OR Micronesia OR Moldova OR Mongolia OR Montenegro OR Morocco OR Mozambique OR Myanmar OR Burmese OR Burma OR Namibia OR Nepal OR Nicaragua OR Niger OR Niue OR Pakistan OR Paraguay OR Peru OR Philippine OR Rwanda OR ‘Sao Tome’ OR Principe OR Senegal OR Serbia OR ‘Sierra Leone’ OR ‘Solomon Island’ OR Somalia OR ‘South Africa’ OR ‘Sri Lanka’ OR ‘St Lucia’ OR ‘Saint Lucia’ OR ‘St Vincent’ OR ‘Saint Vincent’ OR Grenade OR Sudan OR Suriname OR Swaziland OR Eswatini OR Syria OR Tajik OR Tanzania OR Zanzibar OR Thai OR Timor OR Togo OR Tonga OR Tunisia OR Turkey OR Turkish OR Turkmen OR Tuvalu OR Uganda OR Ukraine OR Uzbek OR Vanuatu OR Venezuela OR Vietnam OR ‘Vietnam’ OR ‘West Bank’ OR Gaza OR Palestine OR Yemen OR Zambia OR Zimbabwe OR ‘Western Sahara’ OR Argentina OR Russia |
